# SARS-CoV-2-Triggered Hemophagocytic Lymphohistiocytosis with Complications of Posterior Reversible Encephalopathy Syndrome

**DOI:** 10.1155/2024/8829060

**Published:** 2024-07-30

**Authors:** Ross M. Perry, Scott D. Casey, Alex Q. Lee, Sylvia P. Bowditch, Mary A. Rasmussen, Viyeka Sethi, Arun R. Panigrahi

**Affiliations:** ^1^ School of Medicine University of California, Davis, Sacramento, California, USA; ^2^ Department of Emergency Medicine University of California, Davis, Sacramento, California, USA; ^3^ Department of Pediatrics University of California, Davis, Sacramento, California, USA; ^4^ Department of Pediatric Critical Care Medicine University of California, Davis, Sacramento, California, USA; ^5^ Department of Pediatric Hematology and Oncology University of California, Davis, Sacramento, California, USA

## Abstract

In this article, we describe a novel case of SARS-CoV-2-associated-hemophagocytic lymphohistiocytosis (HLH) complicated by posterior reversible encephalopathy syndrome (PRES). Initially diagnosed with multisystem inflammatory response in children (MIS-C), the patient received a large corticosteroid dose days before the onset of neurological symptoms. After developing PRES, the patient was treated with antihypertensives, antiepileptics, dexamethasone, and anakinra, leading to neurologic normalization. We propose that given the challenging diagnostic picture of PRES developing in patients with HLH or MIS-C, institutionalized standards for blood pressure management during corticosteroid induction may significantly improve outcomes in patients being treated for hyperinflammatory syndromes who develop neurological symptoms.

## 1. Introduction

The severe acute respiratory syndrome coronavirus 2 (SARS-CoV-2) pandemic has increased hospital admissions for pediatric hyperinflammatory syndromes including multisystem inflammatory syndrome in children (MIS-C) and, even more rarely, hemophagocytic lymphohistiocytosis (HLH) [1]. High dose corticosteroids, a mainstay of treatment for both HLH and MIS-C [1, 2], may induce hypertensive urgency; therefore, increasing the risk of posterior reversible encephalopathy syndrome (PRES), a transient clinicoradiographic syndrome is thought to be secondary to dysregulation of cerebral blood flow [3].

We present this case as evidence that during the treatment for MIS-C, the clinical picture may rapidly evolve to HLH and even PRES and prompt pharmacological adjustments may minimize morbidity in patients who develop central nervous system (CNS) symptoms.

## 2. Results

### 2.1. Case Description

The patient is a fully immunized, previously healthy 6-year-old female who presented with a 2-month history of relapsing and remitting fevers and lethargy following a PCR-confirmed SARS-CoV-2 infection. On initial evaluation, the patient was afebrile, tachycardic, and hypotensive. Physical exam revealed cracked, erythematous lips and a full body, pruritic rash. She was moaning and minimally verbally responsive. Her hands were cold and pale with delayed capillary refill. Intravenous fluid resuscitation, ceftriaxone, and vancomycin were administered in the emergency department. Within twelve hours, the patient developed a fever of 40°C, signs of multiorgan dysfunction, and fluid-refractory shock requiring norepinephrine.

Laboratory tests revealed a mild leukocytosis, anemia, thrombocytopenia, elevated creatinine, and transaminitis. Additionally, there were elevations in C-reactive protein (CRP) (33 mg/dL), procalcitonin (32.99 ng/mL), ferritin (>20 000 ng/mL), prothrombin time (18.3), and d-dimer (37 178 ng/mL). Fibrinogen was low at 129 mg/dL. Abdominal ultrasound revealed hepatomegaly and borderline splenomegaly. Initial lab tests and x-rays were not concerning for cardiomyopathy or pneumonia, and an echocardiogram confirmed normal heart function.

Meeting criteria for moderate-severe MIS-C (Table 1), the patient began corticosteroid induction therapy. Per institutional standards, she received two doses of 250 mg of methylprednisolone on day one of treatment, followed by 60 mg on days two and three. On treatment day two, intravenous immunoglobulin (IVIG) therapy via was initiated.

With fever, splenomegaly, anemia, thrombocytopenia, hypofibrinogenemia, and hyperferritinemia, the patient also met criteria for HLH (Table 1) with an H-score suggesting a 99%-likelihood of HLH [4]. Cerebrospinal fluid (CSF) cell counts showed histiocytes within normal limits, but confirmatory cytology had not yet resulted.

On treatment day two, the patient's blood pressure stabilized, and norepinephrine was weaned and discontinued. By this time, the patient had defervesced and creatinine had returned to age-appropriate levels.

Despite these improvements, the patient's worsening fibrinogenemia, anemia, and transfusion-resistant thrombocytopenia raised concern for worsening HLH, prompting strong consideration of anakinra. Confirmatory HLH labs such as soluble interleukin-2 receptor (sIL-2R), natural killer cell activity, and bone marrow biopsy were pending, as were the results of CSF cytology.

**Figure 1:**
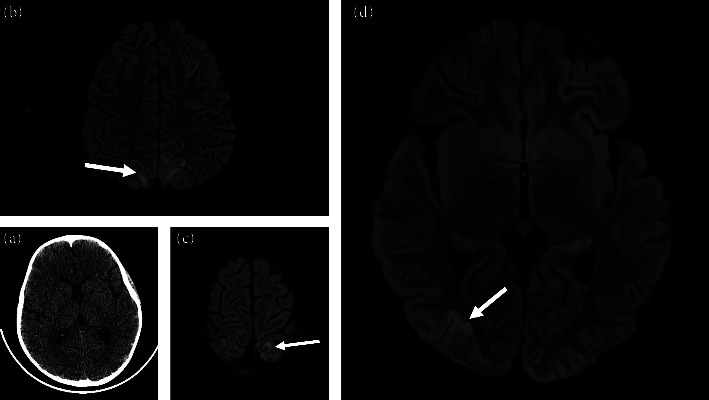
CT and MRI radiographs in a patient presenting with PRES. Following seizure-like activity, an initial CT without contrast (a) showed no acute pathology. A follow-up MRI under sedation (b–d) revealed abnormal, symmetrical signal hyperintensities in the cortex and subcortical white matter of the parietal and occipital lobes on T2-weighted-fluid-attenuated inversion recovery consistent with PRES.

On treatment day 3, the patient experienced a mean blood pressure elevation to the 99^th^-percentile of her age/height-adjusted range (95 mmHg). This was followed by brief hemodynamic desaturation, tonic-clonic movements, and altered mental status. She received hydralazine, levetiracetam, midazolam, and lorazepam and was placed on a nicardipine drip with continuous arterial pressure monitoring via arterial line. Given the concern for CNS HLH, methylprednisolone was changed to dexamethasone, and anakinra was started. An initial head computed topography (CT) scan without contrast showed no acute pathology, while follow-up magnetic resonance imaging (MRI) findings were consistent with PRES (Figure 1).

On treatment day five, the patient self-extubated after a sedative wean. Her neurologic status improved to the point of speaking in short sentences, blood pressure was maintained at age/height-adjusted norms with amlodipine, and ferritin levels continued to downtrend. The patient's neurologic status and ferritin levels returned to the baseline over the following week, and she was discharged home with outpatient hematology follow-up.

Her sIL-2R level eventually resulted, showing a value of 384.636 IU/L from day two of treatment that decreased to 0.8719 IU/L three months later. HLH genetic tests showed two equivocal results: (1) a heterozygous variant in exon 5 of the AP3B1 gene [5] and (2) a heterozygous variant of unknown significance in exon 7 of the LAMP1 gene [6, 7].

## 3. Discussion

This report describes the diagnosis, management, and complications that arose in a case of SARS-CoV-2-triggered-HLH in a previously healthy 6-year-old female with equivocal genetic HLH markers. The patient developed seizure-like activity during the first week of treatment and was diagnosed with PRES via MRI. She quickly returned to neurologic and physiologic baseline after initiation of antiepileptics, antihypertensives, and anakinra.

Her case highlights several complications that may arise in children following SARS-CoV-2 infection. For example, the development of MIS-C symptoms may mask an underlying HLH (Table 1) [1]. While both syndromes can present with fever, shock, elevated CRP, neutropenia, and thrombocytopenia, the presence of hyperferritinemia, anemia, and splenomegaly are more specific to HLH [8]. Henter et al.'s HLH-2004 guidelines may assist in the diagnosis of HLH [2] (Table 1), while Fardet's HScore [4] may aid in predicting the likelihood of HLH while awaiting the results of confirmatory labs such as sIL-2R (Fardet's HScore was originally validated for the diagnosis of “primary HLH.” However, the North American Consortium of Histiocytosis (NACHO) has since recognized that the use of “primary” and “secondary” HLH is an unnecessary and confusing distinction that fails to delineate the difference between “HLH disease” and “HLH disease mimics” [17]. Therefore, we have chosen to omit any discussion of primary and secondary HLH from the current case report). Treatment for MIS-C and HLH involves high-dose corticosteroids and sometimes IVIG [1]. For HLH, early initiation of etoposide, a CD8+ cytotoxic chemotherapy, has been the standard of care [4]. However, recent evidence suggests anakinra, an IL-1 antagonist, is a viable, less chemotoxic alternative that may better attenuate the hyperinflammatory cascade underlying HLH pathophysiology [9].

Mortality is as high as 80% in untreated HLH, and involvement of the CNS portends particularly poor outcomes [10]. Symptoms vary in CNS HLH, but they include seizures, ataxia, and encephalopathic mental status changes similar to those found in PRES [11]. Theoretically, CSF analysis should assist in differentiating these diagnoses. However, as in this case, confirmatory cytology may be slow to return, inciting the need for prompt CNS imaging. Radiographically, PRES often presents with signs of bilateral posterior hemisphere edema on CT or MRI, whereas CNS HLH may show meningeal signs or periventricular hypodense lesions [10, 12]. If PRES is confirmed, treatment includes antiepileptics and blood pressure control [3]. Prognosis of PRES in pediatrics is generally favorable, although some small studies suggest that higher levels of inflammatory markers may be associated with worse outcomes [13].

The current case is unique in in multiple aspects. First, while MIS-C is now a well-documented albeit rare SARS-CoV-2 complication, SARS-CoV-2-related-HLH in children has only once been reported [14]. Second, this case highlights the successful management of HLH with early initiation of anakinra, a relatively new treatment regimen [9]. Finally, the diagnosis and management of PRES during the treatment of SARS-CoV-2-related-HLH is a novel issue that presents several diagnostic challenges.

We considered three diagnostic possibilities following our patient's development of hypertension and seizure: (1) direct CNS HLH, (2) HLH-triggered-PRES, and (3) treatment-triggered PRES. MRI showed bilateral posterior subcortical hyperintensities confirming PRES, and CSF cytology eventually ruled out CNS HLH involvement [10, 11]. COVID-triggered PRES is well-documented in adult literature [15] but thought to be an unlikely etiology given the remote onset of SARS-CoV-2 infection. The mechanism of HLH-associated PRES in our patient is likely multifactorial, involving an HLH-induced proinflammatory state exacerbated by steroid-induced hypertension as previously described [3].

An algorithm to avoid this kind of steroid-induced neurotoxic hypertension is well outlined in the treatment of acute lymphoblastic leukemia [16], but such considerations may not yet be highlighted in institutional treatment guidelines for MIS-C and HLH [1]. As this case suggests, increased attention to hemodynamic monitoring may prevent hypertension-associated PRES during the treatment of hyperinflammatory syndromes.

## 4. Conclusion

This report documents a case of SARS-CoV-2-triggered-HLH in a previously healthy child. Subsequent development of PRES emphasizes the need for institutional guidelines regarding the monitoring and management of acute hypertension and encephalopathy in patients undergoing corticosteroid treatment for MIS-C or HLH.

## Figures and Tables

**Table 1:** Multisystem inflammatory syndrome in children vs. hemophagocytic lymphohistiocytosis diagnostic criteria.

Criteria	MIS-C	HLH^*b*^
Fever (>38°C)	>24 hours	>7 days^*b*^

Severe illness necessitating hospitalization required?	Yes	No^b^

Organ system involvement	≥2 systems affected^*a*^	Splenomegaly^*b*^

Laboratory findings	Elevation in 1 or more of the following: (i) CRP (ii) ESR (iii) Procalcitonin (iv) D-dimer (v) Ferritin (vi) Lactate dehydrogenase (vii) IL-6 (viii) Elevated neutrophils OR Reduction in 1 or more of the following: (i) Lymphocytes (ii) Albumin (i) Fibrinogen AND (i) Evidence of prior SARS-CoV-2 infection	Cytopenia in >2 cell lines^*b*^ (i) Hemoglobin <90 g/L (ii) Platelets <100 × 10^9^/L (iii) Neutrophils <1.0 × 10^9^/L Hypertriglyceridemia OR hypofibrinogenemia^*b*^ (i) Fasting triglycerides ≥3.0 mmol/L (ii) Fibrinogen ≤1.5 g/L Hemophagocytosis on bone marrow biopsy or lymph nodes and no malignancy^*bs*^ Low or absent NK cell activity (according to local laboratory reference)^*b*^ Serum ferritin >500 mg/L^*b*^ Elevated sIL-2R (CD25) > 2.400 IU/L^*b*^ OR (i) Evidence of mutation in a known causative gene

Other criteria	No alternative diagnosis	n/a

A comparison of the diagnostic criteria for multisystem inflammatory syndrome in children [1] and hemophagocytic lymphohistiocytosis [2]. MIS-C, multisystem inflammatory response in children; HLH, hemophagocytic lymphohistiocytosis; CRP, C-reactive protein; ESR, erythrocyte sedimentation rate; IL-6, interleukin-6; SARS-CoV-2, severe acute respiratory syndrome coronavirus 2; NK cell activity, natural killer cell activity; sIL-2R, soluble interleukin-2 receptor. ^*a*^Systems include cardiac, renal, respiratory, hematologic, gastrointestinal, dermatological, and neurological. ^*b*^At least five of the eight criteria marked with “*b*” are required for diagnosis of HLH [2].

## Data Availability

The underlying data lie in the electronic health record of the University of California, Davis Medical Center.

## Abbreviations

SARS-CoV-2: Severe acute respiratory syndrome coronavirus 2
HLH: Hemophagocytic lymphohistiocytosis
PRES: Posterior reversible encephalopathy syndrome
MIS-C: Multisystem inflammatory syndrome in children
CNS: Central nervous system
IVIG: Intravenous immunoglobulin
CRP: C-reactive protein
CSF: Cerebrospinal fluid
CT: Computed tomography
MRI: Magnetic resonance imaging.

## References

[1] Nakra N., Blumberg D., Herrera-Guerra A., Lakshminrusimha S. (2020). Multi-system inflammatory syndrome in children (MIS-C) following SARS-CoV-2 infection: review of clinical presentation, hypothetical pathogenesis, and proposed management. *Children*.

[2] Henter J., Horne A., Aricó M. (2007). HLH‐2004: diagnostic and therapeutic guidelines for hemophagocytic lymphohistiocytosis. *Pediatric Blood and Cancer*.

[3] Lee G., Lee S. E., Ryu K., Yoo E. S. (2013). Posterior reversible encephalopathy syndrome in pediatric patients undergoing treatment for hemophagocytic lymphohistiocytosis: clinical outcomes and putative risk factors. *Blood Research*.

[4] Fardet L., Galicier L., Lambotte O. (2014). Development and validation of the HScore, a score for the diagnosis of reactive hemophagocytic syndrome. *Arthritis and Rheumatology*.

[5] Jung J. (2006). Identification of a homozygous deletion in the AP3B1 gene causing hermansky-pudlak syndrome, type 2. *Blood*.

[6] Miao Y., Zhu H., Qiao C. (2019). Pathogenic gene mutations or variants identified by targeted gene sequencing in adults with hemophagocytic lymphohistiocytosis. *Frontiers in Immunology*.

[7] Rubin T. S., Zhang K., Gifford C. (2017). Perforin and CD107a testing is superior to NK cell function testing for screening patients for genetic HLH. *Blood*.

[8] Gupta S., Weitzman S. (2010). Primary and secondary hemophagocytic lymphohistiocytosis: clinical features, pathogenesis and therapy. *Expert Review of Clinical Immunology*.

[9] Bami S., Vagrecha A., Soberman D. (2020). The use of anakinra in the treatment of secondary hemophagocytic lymphohistiocytosis. *Pediatric Blood and Cancer*.

[10] Haddad E., Sulis M. L., Jabado N., Blanche S., Fischer A., Tardieu M. (1997). Frequency and severity of central nervous system lesions in hemophagocytic lymphohistiocytosis. *Blood*.

[11] Jovanovic A., Kuzmanovic M., Kravljanac R. (2014). Central nervous system involvement in hemophagocytic lymphohistiocytosis: a single-center experience. *Pediatric Neurology*.

[12] Deiva K., Mahlaoui N., Beaudonnet F. (2012). CNS involvement at the onset of primary hemophagocytic lymphohistiocytosis. *Neurology*.

[13] Chen T. (2020). Childhood posterior reversible encephalopathy syndrome: clinicoradiological characteristics, managements, and outcome. *Frontiers in Pediatrics*.

[14] Kalita P., Laishram D., Dey B., Mishra J., Barman B., Barman H. (2021). Secondary hemophagocytic lymphohistiocytosis in post-COVID-19 patients: a report of two cases. *Cureus*.

[15] Yeahia R., Schefflein J., Chiarolanzio P. (2022). Brain MRI findings in COVID-19 patients with PRES: a systematic review. *Clinical Imaging*.

[16] Murphy L., Maloney K, Gore L., Blanchette E. (2022). Hypertension in pediatric acute lymphoblastic leukemia patients: prevalence, impact, and management strategies. *Integrated Blood Pressure Control*.

[17] Jordan M. B., Allen C. E., Greenberg J. (2019). Challenges in the diagnosis of hemophagocytic lymphohistiocytosis: recommendations from the North American consortium for histiocytosis (NACHO). *Pediatric Blood and Cancer*.

